# Cultural and Linguistic Adaptation of Diagnostic Language Assessments: A Scoping Review

**DOI:** 10.1111/1460-6984.70357

**Published:** 2026-10-18

**Authors:** Mignon Botes, Khetsiwe Masuku, Anniah Mupawose

**Affiliations:** ^1^ Department of Speech‐Language Pathology University of the Witwatersrand Johannesburg South Africa

**Keywords:** cultural adaptation, diagnostic language assessment, linguistic adaptation, multilingual children, scoping review

## Abstract

**Background:**

Diagnostic language assessments are central to identifying developmental language disorder; however, many widely used tools were developed and normed within monolingual Western contexts. Their application in multilingual and multicultural settings raises concerns about linguistic, cultural and psychometric validity. Although adaptation of diagnostic tools is frequently reported, the scope and quality of published adaptation research has not been systematically mapped.

**Aims:**

This scoping review aimed to map the literature on the cultural and linguistic adaptation of diagnostic language assessment tools for multilingual and multicultural child populations, to describe the adaptation and validation approaches used, and to identify gaps relevant to future diagnostic tool development.

**Methods and Procedures:**

A scoping review was conducted following the Arksey and O'Malley framework and reported in line with PRISMA‐ScR guidelines. Searches were conducted across four electronic databases for peer‐reviewed studies published between 2014 and 2025. Studies (*n* = 14) were included if they reported the translation, cultural adaptation, or contextual modification of diagnostic language assessments for children aged 3–12 years. Data were charted on study characteristics, assessment tools, adaptation frameworks, validation methods and reported challenges.

**Outcomes and Results:**

Fourteen studies met inclusion criteria, representing 10 countries. Adapted tools assessed a range of language domains. Most studies reported multi‐step adaptation processes and referenced established international frameworks; however, the depth of cultural adaptation and the rigour of psychometric validation varied substantially.

**Conclusions and Implications:**

Diagnostic language assessment adaptation research remains fragmented and methodologically inconsistent. The limited evidence base across diverse multilingual settings highlights an urgent need for contextually grounded, psychometrically robust diagnostic tools to support equitable assessment in multilingual settings.

**WHAT THIS PAPER ADDS:**

*What is already known on this subject*

- Diagnostic language assessments are often developed in monolingual Western contexts and may not be valid for multilingual children.
- Translation and cultural adaptation of assessments are increasingly reported, but practices vary widely.

*What this paper adds to existing knowledge*

- This review provides a systematic mapping of diagnostic language assessment adaptations, highlighting substantial variation in adaptation depth and validation quality.
- Only 14 studies across 10 countries were identified, indicating that the evidence base for culturally and linguistically adapted diagnostic language assessments remains limited and geographically fragmented.

*What are the potential or actual clinical implications of this work?*

- Clinicians should exercise caution when using translated diagnostic tools and adopt multi‐method, culturally responsive assessment practices.
- The findings provide a framework to guide future diagnostic tool adaptation and development in multilingual contexts.

## 1 Introduction

The accurate diagnosis of language impairment relies on the use of valid and reliable assessment tools that can capture a child's receptive, expressive, phonological and pragmatic language abilities (Camarata and Nelson 2002). Standardised diagnostic assessments play a central role in speech‐language pathology, guiding clinical decision‐making and informing appropriate and effective intervention (Denman et al. 2017). However, the validity of these tools depends on their cultural and linguistic appropriateness for the population being assessed (Carter et al. 2005; Mdlalo et al. 2019). When applied to multilingual or culturally diverse children using norms developed in monolingual, Western contexts, diagnostic tools risk misrepresenting ability, inflating impairment or obscuring true difficulty. These concerns are well documented internationally (Grech and Dodd 2011; Freeman and Schroeder 2022; Carter et al. 2005) and are particularly relevant in multilingual and multicultural contexts where clinicians frequently assess children from diverse linguistic and cultural backgrounds.

Multilingual and multicultural child populations challenge traditional assumptions about typical language development and standardised assessment practices. Many diagnostic tools, including widely used instruments such as the Clinical Evaluation of Language Fundamentals (CELF), Preschool Language Scale (PLS) and Peabody Picture Vocabulary Test (PPVT), were developed, standardised, and normed on homogeneous English‐speaking samples (Campbell and Dommestrup 2010; Coret and McCrimmon 2015). Applying these tools to children whose linguistic experiences differ substantially from the original normative groups introduces a risk of both under‐ and over‐diagnosis (Freeman and Schroeder 2022; Grech and Dodd 2011). Even when clinicians adapt tools informally—by translating items, modifying test stimuli or adjusting scoring—such adaptations are rarely systematic, validated or documented. As a result, their psychometric properties become uncertain, and clinical interpretation becomes highly variable.

Challenges associated with culturally and linguistically appropriate assessment are particularly evident in African countries that are among the most linguistically diverse countries, such as Ghana, Kenya, Nigeria and South Africa (Bampoe et al. 2025; Mdlalo et al. 2019). Linguistic profiles vary substantially across regions, communities and households, with frequent code‐switching and complex language exposure patterns (Southwood 2014). Despite this diversity, the speech‐language pathology profession continues to rely largely on imported, Western‐developed diagnostic tools (Van Dulm and Southwood 2013). For example, the majority of available tools have not been translated, culturally adapted or normed for South African children, and recent evidence indicates that only a small number of child language assessments in South Africa have undergone psychometric evaluation—none of which are fully normed or validated for diagnostic decision‐making across languages (Southwood et al. 2025). As highlighted in national guidelines, culturally and linguistically responsive assessment remains an ethical imperative (HPCSA 2019); yet systematic adaptation research is notably lacking.

The challenges illustrated by the African context are not unique and are experienced across multilingual and multicultural contexts globally. Existing reviews have mapped the translation of developmental screeners (El‐Behadli et al. 2015) or have examined culturally relevant assessment practices for Indigenous populations (Higgins and Lefebvre 2024), but there is very limited reviews to date has synthesised research on the adaptation of *diagnostic language assessment tools* for multilingual and multicultural populations. Importantly, diagnostic tools differ substantially from screeners in purpose, complexity and psychometric demands, meaning that evidence from screening translations cannot be assumed to generalise to diagnostic contexts. Moreover, existing research varies widely in how adaptation is conceptualised and implemented—ranging from direct translations to complex cultural adaptations involving community consultation, back‐translation and psychometric revalidation.

A preliminary scan of the literature conducted for this review further highlighted substantial variability in adaptation processes globally. This variability underscores a critical need to understand how adaptations are being conducted internationally and what models or methods might guide future diagnostic tool development in multilingual and culturally diverse contexts. A scoping review is therefore needed to map the extent, nature, and methodological characteristics of existing research on adapted diagnostic language assessment tools.

The aim of this scoping review is to systematically chart the literature on the adaptation of diagnostic language assessment tools for multilingual and multicultural populations, identify key characteristics of adaptation processes, and highlight gaps that warrant further investigation. In doing so, the review seeks to provide an evidence‐based foundation for future adaptation and validation work in multilingual and culturally diverse settings internationally, with implications for contexts such as South Africa.

## 2 Method

### 2.1 Study Design

This scoping review was conducted following the methodological framework proposed by Arksey and O'Malley (2005) and later refined by Levac et al. (2010), as well as guidance from the Joanna Briggs Institute (Peters et al. 2015). Reporting adhered to the Preferred Reporting Items for Systematic Reviews and Meta‐Analyses extension for Scoping Reviews (PRISMA‐ScR) (Tricco et al. 2018). The methodological process included identifying the research question, identifying and selecting relevant studies, charting the data, and collating and summarising the findings. No protocol for this scoping review was formally registered. The review followed established scoping review guidance. No formal critical appraisal was conducted, as the aim was to map the extent and nature of the evidence (Arksey and O'Malley 2005; Peters et al. 2015).

### 2.2 Research Question

The review was guided by the question: What diagnostic language assessment tools have been adapted for multilingual and multicultural populations, and what translation or cultural adaptation methods were employed to ensure linguistic and contextual relevance? Sub‐aims were to identify the characteristics of adaptation processes, challenges and facilitators encountered during adaptation, and the validation or psychometric approaches used to evaluate adapted tools. For the purposes of this review, adaptation was broadly defined as any reported process of translation, cultural modification, contextual modification or psychometric validation undertaken to improve the linguistic or cultural appropriateness of a diagnostic language assessment tool for a new population.

### 2.3 Eligibility Criteria

Eligibility criteria were informed by the Population–Concept–Context (PCC) framework (Peters et al. 2015). The population included studies involving human participants aged 3–12 years. This age range was selected to align with the period in which formal diagnostic language assessments are most commonly administered in speech‐language pathology practice, spanning preschool to late primary school years where diagnostic differentiation of language disorders is most critical. Studies focusing exclusively on children younger than 3 years were excluded because this population aligns with early communication intervention rather than diagnostic language assessment. Studies including participants older than 12 years were excluded to maintain focus on childhood diagnostic assessment, as language assessment practices and tools differ substantially in adolescent populations. Studies were included where the reported sample substantially overlapped with the 3–12‐year age range, even if the total sample extended slightly beyond these limits. This approach ensured inclusion of relevant adaptation studies while maintaining conceptual alignment with the target population. The concept comprised studies describing the translation, cultural adaptation, contextual modification or validation of diagnostic language assessment tools. Eligible adaptations included linguistic translation, cultural modification or hybrid processes. Studies that primarily focused on validation were included where an adaptation or modification of the tool was explicitly described, recognising that adaptation and validation processes are often closely linked in applied assessment research. Screening tools, developmental checklists, autism spectrum disorder–specific observation measures, and parent‐only report instruments were excluded. The context included studies conducted in any country or linguistic setting, with particular consideration of multilingual and multicultural populations. The time frame (2014–2025) was selected to capture contemporary adaptation practices and current methodological approaches, particularly reflecting increased global attention to culturally and linguistically responsive assessment over the past decade. The review was limited to English‐language publications due to feasibility constraints; however, it is acknowledged that this may have resulted in the exclusion of relevant studies published in other languages, particularly given the cross‐cultural nature of the topic. Grey literature, including theses and reports, was excluded to ensure that included studies met peer‐review standards and provided sufficient methodological detail for analysis. While this may have limited the inclusion of emerging or context‐specific work, the decision was made to prioritise methodological transparency and quality.

### 2.4 Search Strategy

A three‐step search strategy consistent with JBI guidelines was implemented (Peters et al. 2015). First, an initial, limited search was conducted in PubMed to refine keywords, indexing terms and search syntax. Narrow combinations of search terms yielded very few relevant studies, indicating that adaptation‐focused research is inconsistently indexed and dispersed across disciplines. Second, a broader and iteratively refined search strategy was developed and applied across MEDLINE Ultimate, CINAHL Ultimate, ERIC and PubMed. Broader combinations of terms retrieved a larger and more representative set of adaptation‐related literature. The final search string combined controlled vocabulary and free‐text terms relating to language assessment and adaptation: (‘language assess*’ OR ‘language test*’ OR ‘speech assess*’ OR ‘speech test*’) AND (‘adapt*’ OR ‘translat*’) AND child. The inclusion of both ‘speech’ and ‘language’ terms was intentional to ensure comprehensive capture of relevant studies, as terminology is used inconsistently across disciplines and some diagnostic language tools are indexed under speech‐related terms. Filters were applied to retrieve English‐language, peer‐reviewed academic articles published between 2014 and 2025, with full‐text availability and participants aged 3–12 years. Full database‐specific search strategies are available from the authors upon reasonable request. Finally, reference lists of all included studies were screened to identify additional eligible sources.

All retrieved records were uploaded to Rayyan, a web‐based review management platform (Ouzzani et al. 2016). Duplicates were removed. Two reviewers independently screened titles and abstracts in Rayyan with blinding enabled, and conflicts were resolved through consensus or consultation with a third reviewer when needed. The same blinded process was followed for full‐text review, with reasons for exclusion documented. The study selection process was summarised using a PRISMA‐ScR flow diagram (see Figure 1).

**FIGURE 1:**
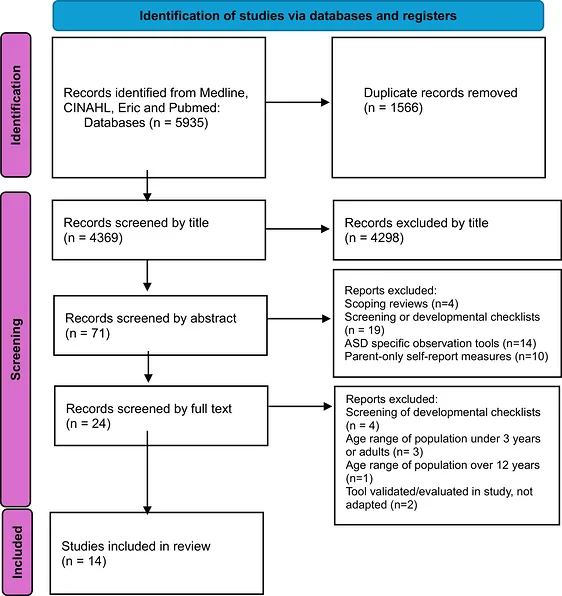
PRISMA‐ScR flow diagram.

### 2.5 Data Extraction

Data were charted using a structured Microsoft Excel extraction tool developed for this review. Extracted information included study identification and geographic details, participant characteristics, diagnostic tool information (name, linguistic origin, domains assessed), type and method of adaptation, cultural or contextual modifications, translation procedures, validation and psychometric evidence, implementation considerations, reported challenges and facilitators, and author recommendations. Data extraction was completed by the primary reviewer and independently verified by a second reviewer. Consistent with scoping review methodology, the extraction tool was refined iteratively as familiarity with the literature increased; any amendments were documented.

A PRISMA‐ScR flow diagram, presented in Figure 1, was used to present the number of records identified, screened, included and excluded, along with reasons for exclusion, adapted from Page et al. (2021) (Figure 1).

## 3 Results

### 3.1 Study Selection

All search results were loaded into Rayyan (Ouzzani et al. 2016) for blinded screening. A total of 5935 records were identified across databases. After removing 1566 duplicates, 4369 titles were screened, of which 4298 were excluded as clearly irrelevant. Seventy‐one abstracts were reviewed and 47 were excluded (e.g., scoping reviews, screening or ASD‐specific tools, parent‐report studies). Twenty‐four full‐text articles were assessed for eligibility; 10 were excluded because they focused on screening tools, did not report an adaptation process, or fell outside the target age range, leaving 14 studies in the final review. The study selection process is presented in Figure 1. Studies with mixed‐age samples were included if a substantial proportion of participants fell within the predefined age range (3–12 years), or if data for this age group could be clearly extracted.

### 3.2 Characteristics of Included Studies

The 14 included studies were published between 2014 and 2025 and originated from 10 countries. Adaptations were concentrated in Brazil (Fortunato Queiroz et al. 2023; Lindau et al. 2014; Rossi et al. 2016; da Silva et al. 2023) and Turkey (Karamete and Toğram 2025; Saban‐Dülger et al. 2022; Sahli and Belgin 2017). Additional studies came from Jordan (Alkhamra and Al‐Jazi 2016), Guatemala (Connery et al. 2019), Kenya (Knauer et al. 2019), Iran (Salmani et al. 2022), Madagascar (Weber et al. 2015), the United Kingdom (Pennington et al. 2025) and Saudi Arabia (Yousef et al. 2024). Several studies combined adaptation and validation processes, reflecting the close integration of these stages in applied assessment research.

Samples ranged from 1;6 to 16 years; however, all included studies contained participants within the target age range (3–12 years), consistent with the review's inclusion criteria. On the whole, samples comprised typically developing (TD) children, with some studies including clinical groups such as children with language disorders (Sahli and Belgin 2017), children using Augmentative or Alternative Communication (AAC) or with low motor function (Pennington et al. 2025), children with suspected Childhood Apraxia Speech (CAS) or severe Speech Sound Disorders (SSD) (Karamete and Toğram 2025), and children with hearing impairment (Yousef et al. 2024). Overall, the field remains weighted towards TD samples, with fewer adaptations validated in clinical populations.

Most adaptations were developed and tested with typically developing children, often as a first step towards later clinical use. The Preschool Language Assessment Instrument–Second Edition (PLAI‐2), the Test of Narrative Language—Brazilian Portuguese version (TNL‐BP), the New Reynell Developmental Language Scales (NRDLS), the Peabody Picture Vocabulary Test–Third Edition (PPVT‐III) and the Persian Language Assessment, Remediation and Screening Procedure (P‐LARSP) were all initially adapted and piloted with typically developing samples (Lindau et al. 2014; Rossi et al. 2016; Fortunato Queiroz et al. 2023; Weber et al. 2015; Salmani et al. 2022). A smaller number of studies incorporated clinical or high‐risk groups during validation. For example, the Turkish Preschool Language Scale–5 (TPLS‐5) included children with language impairments alongside typically developing peers (Sahli and Belgin 2017), the UK Computer‐Based Instrument for Low Motor Language Testing (C‐BiLLT‐UK) included children with complex motor profiles using augmentative and alternative communication access methods (Pennington et al. 2025), the Dynamic Evaluation of Motor Speech Skill—Turkish version (DEMSS‐TR) targeted children with childhood apraxia of speech and severe speech sound disorders (Karamete and Toğram 2025), and the Saudi Paediatric Speech Intelligibility Test (SPSI) focused on children with hearing loss (Yousef et al. 2024). This pattern reflects a normative trajectory in adaptation work: early pilots with TD children to establish feasibility and age trends, followed by gradual extension to clinical cohorts. However, few studies reported comprehensive diagnostic accuracy indices (e.g., sensitivity/specificity, ROC cut‐offs) in clinical samples, limiting conclusions about the diagnostic performance of the adapted tools.

Table 1 gives an overview of the year of publication, country, age range of participants and sample size used in the study.

**TABLE 1:** Characteristics of the studies.

Study	Year	Country	Sample (*n*, age range)
Alkhamra and Al‐Jazi	2016	Jordan	*n* = 432, 3–12 years
Connery et al.	2019	Guatemala	*n* = 157, 42–68 months
Fortunato Queiroz et al.	2023	Brazil	*n* = 8, 2–7 years
Karamete and Toğram	2025	Turkey	*n* = 82, 3–6 years
Knauer et al.	2019	Kenya	*n* = 505, 2–6 years
Lindau et al.	2014	Brazil	*n* = 354, 3–5; 11 years
Pennington et al.	2025	UK	*n* = 448, 1;6–12 years
Rossi et al.	2016	Brazil	*n* = 56, 5–11 years
Saban‐Dülger et al.	2022	Turkey	*n* = 80, 2;0–5;11 years
Sahli and Belgin	2017	Turkey	*n* = 1320, 0–7;11 years
Salmani et al.	2022	Iran	*n* = 120, 2–5 years
da Silva et al.	2023	Brazil	*n* = 100, 4–16 years
Weber et al.	2015	Madagascar	*n* = 1372, 3–10 years
Yousef et al.	2024	Saudi Arabia	*n* = 100, 7–11 years

### 3.3 Diagnostic Tools and Domains Adapted

Each study focused on a specific diagnostic instrument, with no single tool dominating the corpus. Comprehensive language batteries included the Preschool Language Scale–5 (TPLS‐5) in Turkish (Sahli and Belgin 2017), the New Reynell Developmental Language Scales (NRDLS) in Brazilian Portuguese (Fortunato Queiroz et al. 2023), the Preschool Language Assessment Instrument—Second Edition (PLAI‐2) in Brazilian Portuguese (Lindau et al. 2014), and the UK adaptation of the Computer‐Based Instrument for Low Motor Language Testing (C‐BiLLT) for low‐motor access (Pennington et al. 2025).

Single‐domain language measures targeted a range of constructs. Receptive vocabulary and lexical comprehension were adapted using the Peabody Picture Vocabulary Test–Third Edition (PPVT–III) in Madagascar (Weber et al. 2015). Narrative skills were assessed through the Test of Narrative Language—Brazilian Portuguese version (TNL–BP) (Rossi et al. 2016). Morphosyntax and grammatical profiling were addressed by the Persian Language Assessment, Remediation, and Screening Procedure (P‐LARSP) (Salmani et al. 2022) and Turkish versions of Developmental Sentence Scoring (DSS‐TR) and the Index of Productive Syntax (IPSyn‐TR) (Saban‐Dülger et al. 2022). Token‐based receptive linguistic comprehension was examined in the Arabic Token Test for Children (A‐TTFC) (Alkhamra and Al‐Jazi 2016).

Two further adaptations addressed adjacent but clinically relevant constructs: speech‐motor planning and Childhood Apraxia of Speech diagnosis through the Dynamic Evaluation of Motor Speech Skill—Turkish version (DEMSS‐TR) (Karamete and Toğram 2025), and speech intelligibility in children with hearing loss via the Saudi Paediatric Speech Intelligibility Test (SPSI) (Yousef et al. 2024). Together, these studies show that adaptation efforts are fragmented and highly context‐specific, with each team tailoring instruments to their own clinical and linguistic environment rather than converging on a small set of global standards. Table 2 summarises the diagnostic tools, language domains assessed, and adaptation contexts.

**TABLE 2:** Diagnostic tools adapted.

Diagnostic tool	Domain(s)	Original language	Adapted language/Context	Studies
Arabic Token Test for Children (A‐TTFC)	Receptive syntax & semantics	English	Arabic (Jordan)	Alkhamra and Al‐Jazi (2016)
Test de Vocabulario en Imagenes Peabody (TVIP)	Receptive lang & vocabulary	English/Spanish	Guatemala multilingual contexts	Connery et al. (2019)
New Reynell Developmental Language Scales (NRDLS)	Receptive & expressive language	English (UK)	Brazilian Portuguese	Fortunato Queiroz et al. (2023)
Dynamic Evaluation of Motor Speech Skill—Turkish Version (DEMSS‐TR)	Speech‐motor planning	English	Turkish	Karamete and Toğram (2025)
British Picture Vocabulary Scale (BPVS) and Malawi Developmental Assessment Tool (MDAT)	Vocabulary, developmental language	English	Kenyan/Luo & Malawi	Knauer et al. (2019)
Preschool Language Assessment Instrument—Second Edition (PLAI‐2)	Receptive & expressive pragmatics	English	Brazilian Portuguese	Lindau et al. (2014)
Persian Language Assessment, Remediation, and Screening Procedure (P‐LARSP)	Morphosyntax	English	Persian	Salmani et al. (2022)
UK Computer‐Based Instrument for Low Motor Language Testing (UK C‐BiLLT‐UK)	Low‐motor access receptive language	Dutch	English (UK)	Pennington et al. (2025)
Test of Narrative Language—Brazilian Portuguese version (TNL–BP)	Narrative language	English	Brazilian Portuguese	Rossi et al. (2016)
Developmental Sentence Scoring—Turkish (DSS‐TR) and Index of Productive Syntax—Turkish (IPSyn‐TR)	Morphosyntax	English	Turkish	Saban‐Dülger et al. (2022)
Turkish Preschool Language Scale‐5 (TPLS‐5)	Comprehensive receptive & expressive language	English	Turkish	Sahli and Belgin (2017)
Brazilian Portuguese Children's Communication Checklist–2 (CCC‐2‐BP)	Pragmatics & structural language	English	Brazilian Portuguese	da Silva et al. (2023)
Peabody Picture Vocabulary Test–Third Edition (PPVT–III), Malagasy version	Receptive vocabulary	English	Malagasy	Weber et al. (2015)
Saudi Pediatric Speech Intelligibility (SPSI) Test	Speech intelligibility	English model	Saudi Arabic	Yousef et al. (2024)

### 3.4 Adaptation and Validation Procedures

Across the 14 studies, most authors reported multi‐step adaptation processes combining linguistic and cultural procedures. Nine of the 14 studies explicitly referenced established international frameworks, most commonly the World Health Organization's guidelines for the translation and adaptation of instruments (World Health Organization 2015) and the MAPI linguistic validation process (MAPI Research Trust 2017). These frameworks emphasise structured forward translation, expert panel review, back‐translation, and cognitive pre‐testing to ensure semantic and conceptual equivalence. Several studies also cited the cross‐cultural adaptation guidelines proposed by Beaton et al. (2000) and the psychometric adaptation principles outlined by Hambleton (2004) and the International Test Commission's (ITC) test adaptation guidelines (International Test Commission 2017). The Beaton framework focuses on ensuring semantic, idiomatic, experiential and conceptual equivalence across cultures, while the Hambleton and ITC guidelines emphasise preserving measurement equivalence through rigorous psychometric evaluation. Together, these frameworks shaped the translation, cultural modification and early validation procedures described in the included studies. Although some studies placed greater emphasis on validation, all included studies reported an explicit process of translation, cultural adaptation or contextual modification, which met the inclusion criteria for this review.

For example, the Arabic Token Test for Children drew on WHO (2015) and MAPI recommendations, using forward translation, expert review and pre‐testing (Alkhamra and Al‐Jazi 2016). PLAI‐2 adaptations in Brazil similarly implemented forward translation, expert panel review and pilot testing (Lindau et al. 2014). Turkish adaptations of DSS/IPSyn and the P‐LARSP followed Beaton‐style cross‐cultural adaptation steps (Saban‐Dülger et al. 2022; Salmani et al. 2022), as did the TNL–BP (Rossi et al. 2016) and TPLS‐5 (Sahli and Belgin 2017).

Forward–back translation with expert adjudication was the most common pattern, although reporting depth varied. Several studies described two independent forward translations, reconciliation into a single version, back‐translation into the source language, and review by a multi‐disciplinary panel or the original test author (Rossi et al. 2016; Pennington et al. 2025; Saban‐Dülger et al. 2022; Salmani et al. 2022). Others reported more limited procedures, describing translation by a bilingual speech‐language therapist and informal consultation with colleagues, but without full back‐translation or panel consensus (e.g., early pilots of PLAI‐2 and NRDLS; Lindau et al. 2014; Fortunato Queiroz et al. 2023). One adaptation (DSS‐TR/IPSyn‐TR) explicitly noted that formal translation procedures were not documented in detail in the publication, despite extensive linguistic work (Saban‐Dülger et al. 2022).

Cultural adaptation typically involved lexical and pictorial replacements to ensure that stimuli reflected children's daily experiences. For instance, the Brazilian TNL replaced a ‘McDonald's’ story context with a culturally appropriate ‘Snack Bar’ story (Rossi et al. 2016), and NRDLS and PLAI‐2 adaptations substituted US‐centric holidays and foods with local equivalents (Lindau et al. 2014; Fortunato Queiroz et al. 2023). The Turkish DEMSS‐TR adjusted items to align with Turkish phonotactics and syllable structures (Karamete and Toğram 2025). Weber's PPVT–III adaptation in Madagascar reported substantial dialect‐level adjustment and highlighted differential item functioning for certain lexical items, emphasising the need for careful vetting of vocabulary across dialects (Weber et al. 2015).

Table 3 presents the validation processes used. Validation evidence ranged from basic reliability indices to advanced psychometric modelling. Commonly reported metrics included internal consistency coefficients, test–retest and inter‐rater reliability, and age‐related score gradients (e.g., Alkhamra and Al‐Jazi 2016; Sahli and Belgin 2017; Saban‐Dülger et al. 2022). Several studies conducted factor analyses or convergent validity checks with existing measures (Pennington et al. 2025; Salmani et al. 2022). A small number used more sophisticated methods such as Rasch/Item Response Theory (IRT) analysis, Receiver Operating Characteristic (ROC) curves, or differential item functioning (DIF) analyses to interrogate item behaviour across groups (Weber et al. 2015; Karamete and Toğram 2025; Pennington et al. 2025). Overall, the field appears to be moving from ‘translation plus pilot testing’ toward more rigorous, model‐based validation in newer adaptations.

**TABLE 3:** Adaptation process used.

Study	Translation method(s)	Cultural adaptation procedures	Psychometric evaluation
Alkhamra and Al‐Jazi (2016)	WHO/MAPI approach; forward + expert review	Minor lexical adjustments	Factor analysis; validity
Connery et al. (2019)	Forward translation; expert panel	Adapted procedures for multilingual exposure	Convergent validity
Fortunato Queiroz et al. (2023)	Five‐step process (forward; back‐translation; panel review)	Content substitution for Brazilian context	Content validity; pilot study
Karamete and Toğram (2025)	Forward translation; SLP bilingual team	Adjusted to Turkish phonotactics	Internal reliability; DIF; Rasch
Knauer et al. (2019)	Forward translation; committee review	Cultural relevance adjustments	Internal consistency
Lindau et al. (2014)	Translation with expert review	Minimal cultural edits	Pilot data
Pennington et al. (2025)	International Test Commission guidelines	Pictorial/contextual adjustments	Internal consistency; IRT/Rasch
Rossi et al. (2016)	Forward/back translation	“McDonald's Story” → “Snack Bar Story”	Pilot validity
Saban‐Dülger et al. (2022)	Independent translators + reconciliation	Concept/idiomatic adjustments	Reliability; validity coefficients
Sahli and Belgin (2017)	Forward/back translation	Minor pictorial changes	Normative analysis; reliability
Salmani et al. (2022)	Two bilingual SLP translators	Lexical and conceptual modification	Content validity (CVI)
da Silva et al. (2023)	Translation and linguistic adaptation	Cultural adjustments to items	Mixed reliability measures
Weber et al. (2015)	Full translation of all items	Replacement of culturally unfamiliar items	Rasch analysis; item difficulty
Yousef et al. (2024)	Adaptation from Egyptian → Saudi Arabic	Vocabulary and dialect adjustments	Inter‐rater reliability; pilot

### 3.5 Recurrent Challenges and Facilitators

Authors highlighted several recurrent challenges. Small pilot samples limited the generalisability of early adaptations such as NRDLS and PLAI‐2 (Fortunato Queiroz et al. 2023; Lindau et al. 2014; Knauer et al. 2019). Some teams faced constraints on altering item order or content because of publisher permissions, which affected the PPVT–III adaptation (Weber et al. 2015). Several studies commented on the resource‐intensive nature of transcription and scoring in morphosyntax tools (P‐LARSP, DSS‐TR/IPSyn‐TR; Salmani et al. 2022; Saban‐Dülger et al. 2022). Others noted the difficulty of establishing local norms, recruiting representative samples across regions, or capturing the full complexity of multilingual exposure (Connery et al. 2019; Weber et al. 2015).

Key facilitators included close collaboration with original test developers (Rossi et al. 2016; Pennington et al. 2025; Karamete and Toğram 2025), use of international adaptation frameworks and publisher toolkits (Lindau et al. 2014; Knauer et al. 2019; Sahli and Belgin 2017; Salmani et al. 2022), multi‐disciplinary expert panels, and iterative piloting with children, caregivers and clinicians. Large national samples, as in the TPLS‐5 standardisation (Sahli and Belgin 2017), were particularly important for establishing robust norms. Digital and app‐based formats (C‐BiLLT‐UK, SPSI) were reported to enhance accessibility and engagement, especially for children with motor or hearing impairments (Pennington et al. 2025; Yousef et al. 2024). Table 4 lists the challenges and gaps reported in the studies.

**TABLE 4:** Challenges and gaps reported.

Challenge	Studies reporting it
Lack of normative data	Connery et al. (2019); Lindau et al. (2014); Weber et al. (2015)
Small/non‐representative samples	Fortunato Queiroz et al. (2023); Lindau et al. (2014); Rossi et al. (2016)
Multilingual complexity; dialect variation	Connery et al. (2019); Weber et al. (2015)
Limited cultural appropriateness of original items	Rossi et al. (2016); Karamete and Toğram (2025); Sahli and Belgin (2017)
Resource‐intensive procedures (e.g., transcription)	Saban‐Dülger et al. (2022); Salmani et al. (2022)
Limited validation in clinical populations	Most studies; explicitly Sahli and Belgin (2017); Yousef et al. (2024)
Constraints due to publisher permissions	Weber et al. (2015)
Technology/access barriers	Pennington et al. (2025)
Limited reporting of full adaptation steps	Karamete and Toğram (2025); Lindau et al. (2014)

## 4 Discussion

This scoping review mapped international research on the translation, cultural adaptation and validation of diagnostic language assessment tools for children aged 3–12 years. Despite growing recognition of the need for culturally and linguistically responsive assessment, only 14 eligible studies were identified globally over an 11‐year period, indicating that adaptation research remains limited in both scope and geographical distribution. The findings reveal a fragmented landscape, with substantial variation in the depth of translation, cultural adaptation and psychometric evaluation. Furthermore, the limited geographical distribution of the evidence base suggests that important gaps remain in multilingual and multicultural contexts worldwide.

### 4.1 Fragmented Global Adaptation Landscape

Adaptation activities across the 14 included studies were distributed across 10 countries, with no overlap in the tools selected for adaptation. This fragmentation reflects international concerns that language assessment adaptations are largely driven by isolated national research clusters rather than coordinated developmental pathways (Higgins and Lefebvre 2024; International Test Commission 2017). In contrast to fields where global consensus exists around core assessment instruments, the present review shows a ‘tool‐per‐context’ phenomenon: each team adapted a different instrument, often motivated by local availability rather than by strategic selection of tools with strong cross‐cultural potential. Similar patterns have been noted in cross‐cultural screening and developmental assessment, where local adaptation efforts accumulate but rarely converge into unified practices or shared normative databases (El‐Behadli et al. 2015). This limits opportunities for pooled data, cross‐national validation and the development of robust multilingual norms. The small number of studies identified globally further suggests that adaptation research remains an emerging field rather than an established area of diagnostic language assessment research.

### 4.2 Adaptations Remained Translation‐Heavy Rather Than Culturally Grounded

Although most studies followed structured translation processes—often citing WHO (2015), MAPI (2017), Beaton et al. (2000) or Hambleton (2004) guidelines—cultural adaptation was inconsistently implemented. Translation procedures (forward translation, expert reconciliation, back‐translation and proofing) were widely reported and remain essential for establishing linguistic equivalence. However, only a small subset engaged substantively with cultural equivalence, such as adapting narrative scripts, replacing culturally unfamiliar items or reworking task demands to align with children's lived experiences. This is consistent with longstanding critiques that translation alone does not ensure cultural validity (Carter et al. 2005). The American Psychological Association (2017) emphasise that assessment must account for cultural pathways of communication, interaction and knowledge acquisition. Yet many included adaptations limited cultural work to modifying pictures or changing isolated vocabulary items.

The underrepresentation of culturally grounded modifications is particularly notable given the growing evidence that culture shapes not only vocabulary but also narrative structure, discourse expectations, pragmatic norms and communicative routines (Aririguzoh 2022; Greenfield 1997). The Indigenous assessment literature similarly emphasises that culturally unfamiliar tasks may misrepresent children's capabilities (Higgins and Lefebvre 2024). The findings of this review suggest that global adaptation practices still prioritise surface‐level linguistic equivalence over deeper cultural alignment.

### 4.3 Considerable Variation in Psychometric Rigour Limits Diagnostic Interpretability

The quality and depth of validation procedures varied widely. Some adaptations incorporated rigorous psychometric models such as Rasch analysis, Item Response Theory (IRT), Differential Item Functioning (DIF) analysis or Receiver Operating Characteristic (ROC) curves. These methods offer powerful tools for evaluating whether adapted items behave consistently across linguistic and cultural groups, detecting item bias, examining construct equivalence and identifying optimal diagnostic cut‐offs. Notably, such modern psychometric approaches were applied in only a minority of studies (e.g., Pennington et al. 2025; Weber et al. 2015; Karamete and Toğram 2025).

By contrast, many adaptations relied solely on basic reliability indices or small pilot studies, with limited assessment of diagnostic accuracy. This aligns with broader critiques that multilingual assessment research often stops after translation and initial feasibility (Wang and Li 2020). Without robust evidence of construct validity, invariance across groups and diagnostic precision, adapted tools may not provide reliable information for differential diagnosis. These discrepancies highlight the need for methodologically consistent standards for multilingual adaptation and validation. Moreover, most studies were conducted with typically developing children, with only four studies incorporating clinical or high‐risk populations during adaptation or validation procedures. This limits the ability to determine whether adapted tools can accurately differentiate language impairment from typical variation in multilingual populations and restricts their utility for diagnostic decision‐making.

### 4.4 Implications of Evidence Gaps for Multilingual and Multicultural Contexts

The limited evidence base identified in this review highlights persistent gaps in diagnostic language assessment adaptation across multilingual contexts. These gaps are particularly relevant in countries where substantial linguistic diversity exists alongside limited access to culturally and linguistically appropriate assessment tools. South Africa provides one example, as no diagnostic language assessment adaptations conducted in the country were identified in this review. This gap is consistent with South African research documenting the dominance of English‐normed tools (Southwood et al. 2025), the cultural and linguistic inappropriateness of many imported tests (Mdlalo et al. 2019), and systemic inequities arising when assessment fail to align with children's linguistic repertoires (Khoza‐Shangase and Mophosho 2021). Recent global scholarship has begun articulating pathways for culturally relevant assessment through multiphase, deeply contextualised adaptation processes (Azul and Zimman 2022; Botes et al. 2023; Botes, van der Linde, et al. 2023), but these approaches have not yet been widely extended to formal diagnostic language assessment tools. The challenges identified in this review reflect a broader pattern in multilingual and resource‐constrained contexts, where structural, funding and resource constraints hinder the development of validated assessment tools (Sun and Truong 2025). This highlights the need for coordinated research agendas that prioritise culturally responsive methods and psychometric rigour.

### 4.5 Limitations

This review was limited to peer‐reviewed studies published in English and may therefore have excluded relevant studies reported in other languages. The exclusion of grey literature may also have resulted in the omission of emerging or context‐specific adaptation initiatives. Finally, no formal critical appraisal of included studies was undertaken, consistent with scoping review methodology.

### 4.6 Implications for Clinical Practice in Multilingual and Multicultural Contexts

The findings of this review underscore the urgency of strengthening the evidence base for culturally and linguistically responsive diagnostic assessment. Given the small number of adaptation studies globally and the limited inclusion of clinical populations, clinicians should exercise caution when interpreting scores from translated or adapted tools. Given the wide variability in adaptation quality and the limited psychometric robustness of many translated tools, clinicians should avoid relying solely on standardised scores when making diagnostic decisions. Instead, practice should integrate multiple sources of information, including dynamic assessment, ecologically valid language samples, caregiver and teacher input and culturally grounded observations of communicative behaviour. Clinicians also require training to critically appraise whether an adapted tool is conceptually, culturally, and psychometrically appropriate for a specific child. In contexts where validated tools are limited, practitioners must exercise heightened caution and utilise flexible, multi‐method assessment strategies aligned with relevant professional guidelines and international multicultural practice frameworks. Ultimately, these findings reinforce that valid assessment in multilingual contexts demands clinical judgement informed by cultural knowledge, linguistic expertise and a critical awareness of the limitations of adapted tools.

### 4.7 Future Directions for Culturally Anchored, Psychometrically Robust Adaptation

The identification of only 14 studies across an 11‐year period highlights the limited and emerging nature of diagnostic language assessment adaptation research globally and underscores the need for substantially greater investment in adaptation and validation studies across multilingual contexts. There is a clear need for future research to move beyond translation to develop culturally anchored and psychometrically rigorous diagnostic tools for multilingual populations. Adaptation processes should incorporate deeper cultural considerations—including narrative structures, pragmatic norms and experiential knowledge—while also applying advanced psychometric methodologies such as Rasch modelling, differential item functioning analyses, and invariance testing to ensure measurement equivalence across linguistic groups. Collaborative, multi‐site normative studies across diverse cultural settings would strengthen the generalisability and diagnostic accuracy of adapted tools while reducing the fragmentation currently observed in the literature. Incorporating community participation, transdisciplinary expertise and contextually anchored methodologies will be crucial for creating equitable, valid and trustworthy assessments for multilingual children. These collective efforts can lay the groundwork for diagnostic tools that reflect the lived experiences of diverse language communities and meet international standards for cultural and psychometric integrity.

## 5 Conclusion

This scoping review demonstrates that while diagnostic tool adaptation is occurring internationally, it remains fragmented, translation‐heavy and psychometrically inconsistent. The lack of culturally grounded adaptation and limited validation in clinical populations restrict the diagnostic usefulness of many adapted tools and limit confidence in their ability to accurately distinguish language impairment from typical linguistic variation in multilingual children. Future work should move towards coordinated, culturally anchored and psychometrically robust development pathways that support equitable assessment for multilingual children globally.

## Funding

The authors have nothing to report.

## Conflicts of Interest

The authors declare no conflicts of interest.

## Data Availability

The data that support the findings of this study are available from the corresponding author upon reasonable request.
